# Development and Validation of Novel Senescence-TIME Biomarkers for Predicting the Prognosis and Immunotherapy Responsiveness of SKCM Patients

**DOI:** 10.7150/ijms.127285

**Published:** 2026-02-26

**Authors:** Shiying Fan, Shuya Lu, Zilong Wu, Xu Li, Yisong Gao, Gan Mao, Mao Cai, Tianyu Song, Zuojie Peng, Chong Li, Kaixiong Tao, Wei Li

**Affiliations:** Department of Gastrointestinal Surgery, Union Hospital, Tongji Medical College, Huazhong University of Science and Technology, No. 1277 Jiefang Avenue, Wuhan, 430022, Hubei Province, China.

**Keywords:** skin cutaneous melanoma, tumor immune microenvironment, senescence, immunity, risk score

## Abstract

**Background:**

Increasing evidence indicates that tumor cellular senescence can impair antitumor immunity and promote skin cutaneous melanoma (SKCM) progression. However, effective methods for assessing tumor cellular senescent status and tumor immune microenvironment (TIME) status remain lacking. This study intends to establish a novel Senescence-TIME Risk Score (STIRS) based on senescence and TIME related genes to predict prognosis and immunotherapy responsiveness in SKCM patients, thereby providing new strategies for current clinical personalized treatment.

**Methods:**

We identified distinct senescent microenvironment patterns using t-distributed stochastic neighbor embedding (t-SNE) based on a set of senescence marker genes and predicted the TIME in SKCM using the estimation of stromal and immune cells in malignant tumor tissues using expression data (ESTIMATE) algorithm. Based on this, we divided the SKCM cohort into three groups: low-senescence & high-immunity, high-senescence & low-immunity, and mixed. We analyzed differentially expressed genes (DEGs) between the first two groups. Gene ontology (GO) enrichment analysis, kyoto encyclopedia of genes and genomes (KEGG) enrichment analysis, gene set enrichment analysis (GSEA), and the construction of protein-protein interaction (PPI) network were used to investigate the functional relevance of DEGs. We screened DEGs using least absolute shrinkage and selection operation (LASSO) regression and random forest (RF) algorithm to construct the STIRS. Patients were grouped by the median of STIRS, and differences in expression of immune cells and immune checkpoints between groups were examined. The predictive capability of STIRS for immunotherapy was validated. Finally, we knocked down the core risk gene in the B16 cell line to validate its function.

**Results:**

We identified 994 DEGs predominantly enriched in TIME- and senescence-related pathways. The constructed STIRS comprises six signature genes. Patients in the high-STIRS group exhibited significantly poorer survival than those in the low-STIRS group, and STIRS negatively correlated with immune response and immunotherapy responsiveness. A nomogram integrating STIRS and clinical indicators demonstrated satisfactory predictive performance for SKCM patient prognosis. These findings validate the STIRS model as a reliable independent prognostic indicator. Additionally, knockdown of the core risk gene keratin 17 (KRT17) inhibited the invasion and proliferation of B16 cells, demonstrating the role of KRT17 in the progression of SKCM.

**Conclusion:**

This study proposed a novel STIRS model and selected the core risk gene KRT17 for functional validation, which had potential as a prognostic tool and a guide for creating personalized therapies for SKCM patients.

## Introduction

Skin cutaneous melanoma (SKCM), originating from melanocytes, is one of the most aggressive and lethal skin tumors, with its global incidence increasing annually 1. Early-stage SKCM is amenable to surgical resection, but patients with advanced disease, particularly those with distant metastasis, often require systemic therapies 2. Immune checkpoint inhibitors (ICIs) have become a standard adjuvant treatment to prolong survival in SKCM patients. However, the response to immunotherapy varies significantly among individuals, leading to differing prognoses^3^, ^4^. Therefore, more effective biomarkers are needed to guide therapeutic decisions and accurately predict patient outcomes.

The tumor immune microenvironment (TIME) has emerged as a key area of research in SKCM. TIME is a complex environment consisting of various cellular components—tumor cells, immune cells, stromal cells, and a range of cytokines 5. TIME plays a crucial role in regulating SKCM progression, influencing both the prognosis of patients and the development of novel immunotherapy strategies 6, 7. A comprehensive understanding of TIME remodeling under different conditions is critical for prognosis assessment and personalized treatment approaches.

Epidemiological studies indicate that older individuals constitute a high-risk group for SKCM, suggesting that senescence may influence tumor progression 1, 8. Senescence is a stress-induced state characterized by cell cycle arrest, which leads to significant changes within TIME 9. Notably, senescent cells within the TIME secrete a variety of pro-inflammatory cytokines, growth factors, chemokines, and proteases, collectively referred to as the senescence-associated secretory phenotype (SASP) 10. Initially thought to suppress tumorigenesis, SASP from senescent tumor cells has long-term detrimental effects 11, 12. The aging microenvironment promotes the accumulation of SASP-associated fibroblasts, creating a niche that favors SKCM growth 13. Due to its inflammatory characteristics, SASP profoundly interacts with tumor immunity 14. In late-stage tumors, senescent cells and their SASP suppress immune surveillance, thereby favoring tumor progression 15. Moreover, the senescent microenvironment impairs CD8^+^ T cell function, reducing their ability to control cancer 16. In aged mice, the recruitment of immunosuppressive M2 macrophages and regulatory T cells is significantly increased, further driving the immunosuppressive tumor microenvironment 17. In addition to its effects on immune cells, senescence also influences stromal components, such as cancer-associated fibroblasts (CAFs), which enhance immunosuppression and promote tumor development^18^. Overall, senescence within TIME plays a direct role in regulating tumor progression.

The ESTIMATE algorithm, introduced by Kosuke Yoshihara et al. in 2013, enables the estimation of stromal and immune cell components in tumor tissues based on gene expression data. This algorithm provides valuable insights into the complex immune and stromal components within TIME and has been widely used for prognosis prediction in various cancers 19-21. However, relying solely on the ESTIMATEScore to predict SKCM prognosis is insufficient due to the complexity of TIME. There is a need for additional metrics to enhance prognostic accuracy.

**Figure 1:**
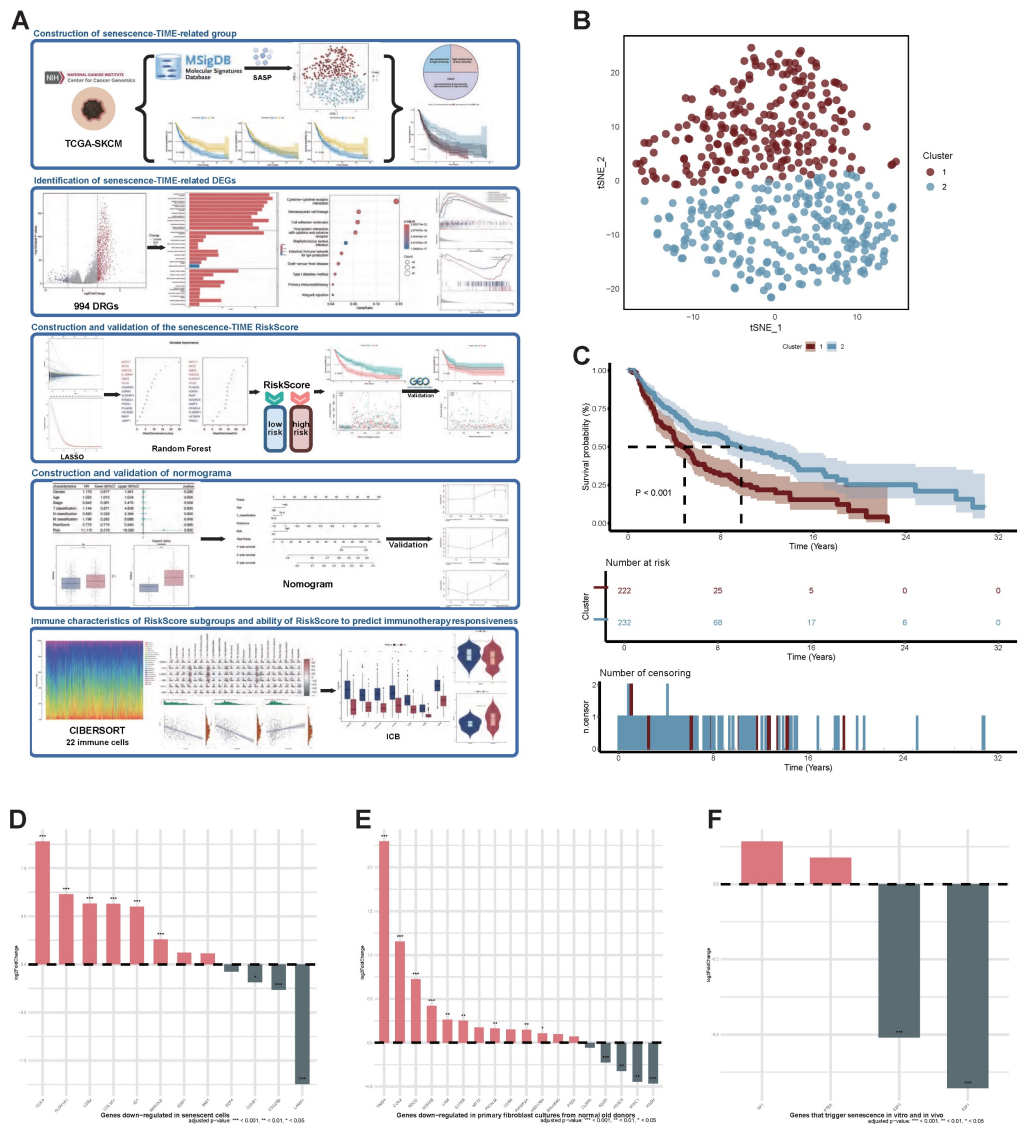
** Identification of senescent status in SKCM Patients.** (A) The flow chart of the bioinformatics analysis procedure (Created in https://BioRender.com). (B) Scatter plot of two distinct clusters identified based on 124 senescence marker genes using the t-SNE algorithm. (C) Kaplan-Meier curves for OS in patients across the two clusters. (D) Expression changes of genes down-regulated in senescent cells across two clusters. FDR-adjusted *p*-value > 0.05; *, FDR-adjusted *p*-value < 0.05; **, FDR-adjusted *p*-value < 0.01; ***, FDR-adjusted *p*-value < 0.001. (E) Expression changes of genes down-regulated in primary fibroblast cultures from normal old donors across two clusters. FDR-adjusted *p*-value > 0.05; *, FDR-adjusted *p*-value < 0.05; **, FDR-adjusted *p*-value < 0.01; ***, FDR-adjusted *p*-value < 0.001. (F) Expression changes of genes that trigger senescence in vitro and in vivo across two clusters. FDR-adjusted *p*-value > 0.05; *, FDR-adjusted *p*-value < 0.05; **, FDR-adjusted *p*-value < 0.01; ***, FDR-adjusted *p*-value < 0.001. **Abbreviations**: SKCM, skin cutaneous melanoma; t-SNE, t-distributed stochastic neighbor embedding; OS, overall survival.

In this study, we constructed and validated a novel Senescence-TIME Risk Score (STIRS), based on the RNA sequencing (RNA-seq) data and clinical information from the cancer genome atlas program (TCGA) online database, which demonstrates robust and accurate performance in predicting SKCM patient prognosis and immunotherapy response (Figure 1A). The STIRS offers promising potential as a biomarker to guide clinical decision-making and optimize precision treatment strategies for SKCM. We also selected the core risk gene keratin 17 (KRT17) for cellular experiments to verify its function, which strengthened the reliability of the bioinformatics analysis results.

## Methods and materials

### Data collection and processing

RNA-seq data and clinical information from 473 SKCM patients were downloaded from TCGA. Data from 469 cases of both primary and metastatic tumors were retained. The gene expression omnibus (GEO) database was the source of the GSE65904 and GSE115821 datasets. Transcripts per million (TPM) underwent log2 conversion.

### Survival analysis

The R package “survminer” was utilized to do survival analysis. The Kaplan-Meier method was used to plot survival curves, and log-rank tests were used to determine statistical significance: *p*-value < 0.05 was deemed significant.

### Identification of senescent status

The t-distributed stochastic neighbor embedding (t-SNE) algorithm was applied to infer the senescent status of tumor tissues in the TCGA-SKCM dataset 22. The t-SNE is a nonparametric, unsupervised method that divides patients into distinct clusters based on given features 22. The senescence signature gene set (genes upregulated in senescent cells: SASP), comprising 124 genes, was downloaded from MsigDB. Groups were classified as “mild senescent microenvironment” or “severe senescent microenvironment” based on survival differences between clusters. Further analysis examined expression changes in other genes involved in senescence-related regulatory processes between the two groups, including 12 genes down-regulated in senescent cells, 18 genes down-regulated in primary fibroblast cultures from normal old donors, and 4 genes that trigger senescence in vitro and in vivo, to explore the relationship between the divided groups and senescence.

### Identification of TIME status

The R package “estimate” was used to calculate the ImmuneScore, StromalScore, and ESTIMATEScore for 469 tumor samples. Subsequently, the 453 samples with survival data were divided into high-score and low-score groups based on the median score, and survival differences between the two groups were compared.

### Identification of senescence-TIME-related groups and differentially expressed genes (DEGs) between groups

Further combining the aforementioned senescent and TIME status into a two-dimensional metric, patients were categorized into three groups: low-senescence & high-immunity, high-senescence & low-immunity, and mixed. The gene expression profiles of the low-senescence & high-immunity group and the high-senescence & low-immunity group were compared using the R package “DESeq2” to identify 994 senescence-TIME-related DEGs (|log2FC| > 2, FDR-adjusted *p*-value < 0.001).

### Gene ontology (GO) enrichment analysis, kyoto encyclopedia of genes and genomes (KEGG) enrichment analysis, gene set enrichment analysis (GSEA), and the construction of protein-protein interaction (PPI) network

GO enrichment analysis, KEGG enrichment analysis, and GSEA were performed using the R package “clusterProfiler” to explore the functions of senescence-TIME-related genes. The PPI network was constructed using the STRING database and reconstructed using Cytoscape version 3.10.3.

### Establishment and validation of STIRS

The least absolute shrinkage and selection operation (LASSO) regression was applied using the R package “glmnet” to screen 15 genes from 994 DEGs. To further simplify the model, random forest (RF) algorithm was conducted using the R package “randomForest”. Ultimately, the top 6 genes with the highest mean decrease accuracy and mean decrease gini were selected as the final key genes for constructing the STIRS. STIRS = ∑(coef × mRNA expression), where coef represents the coefficients derived from LASSO. Using the R package “pROC”, we calculated the area under the receiver operator characteristic (ROC) curve (AUC). STIRS was validated using the external dataset GSE65904.

### Construction and validation of the prognostic nomogram for SKCM

Univariate and multivariate Cox regression analyses identified indicators associated with overall survival (OS). Using R package “survival” and “rms”, nomogram was constructed based on STIRS and relevant clinical parameters to estimate 1-, 3-, and 5-year survival probabilities. The calibration curve was used to assess the model's performance.

### Immune infiltration analysis

Cell-type identification by estimating relative subsets of RNA transcripts (CIBERSORT) analysis was performed using the R package “CIBERSORT” to assess the percentage of 22 immune cell types in each SKCM patient 23. Single sample GSEA (ssGSEA) was conducted using the R package “GSVA” to evaluate the expression of 28 immune cell types in SKCM patients.

### Immune checkpoint expression and immunotherapy responsiveness analysis

Samples were stratified into high-STIRS and low-STIRS groups based on the median STIRS score. Expression levels of immune checkpoints, including programmed cell death protein 1 (PD-1), programmed cell death ligand 1 (PD-L1), and cytotoxic T lymphocyte-associated antigen-4 (CTLA-4), were quantified in both groups. Additionally, the microsatellite instability (MSI) score in the tumor immune dysfunction and exclusion (TIDE) system was calculated using the online platform (http://tide.dfci.harvard.edu/) to assess potential response to ICIs 24. Differences in the response to ICIs were compared between the high-STIRS and low-STIRS groups in the GSE115821 dataset 21.

### Correlation of signature genes with senescence- and immunity-related genes

The expression profiles of signature genes constituting STIRS, as well as senescence- and immunity-related genes in SKCM samples were extracted from the dataset. Spearman's rank correlation analysis was performed to evaluate the correlation between the expression of signature genes and the expression of senescence- and immunity-related genes.

### Cell culture

The mouse melanoma cell line B16 and human embryonic kidney cell line 293T were obtained from Shanghai Zhong Qiao Xin Zhou Biotechnology Co., Ltd., China. Cells were cultured in DMEM (Thermo Fisher Scientific, USA) supplemented with 10% fetal bovine serum (CellMaxcell Technology, China) and maintained at 37℃ in a humidified incubator with 5% CO_2_.

### shKRT17 plasmid construction and lentivirus packaging

The shKRT17 plasmid and shNC plasmid (Corues Biotechnology, China) were co-transfected with pSPAX2 and pMD2.G plasmids at a specific ratio into 293T cells using CW Plasmid Transfection Reagent (CWBIO, China) to package lentivirus. Subsequently, B16 cells were infected with this lentiviral-mediated RNA interference system to achieve stable transfection. A 1μg/mL puromycin selection was used to screen for resistant transfected clones. Transfection efficiency was confirmed by quantitative reverse transcription polymerase chain reaction (RT-qPCR). The shRNA sequence targeting KRT17 was listed in Table S1 of the supplementary file.

### RT-qPCR

RT-qPCR was employed to detect the expression of total RNA extracted from cultured cells. Total RNA was isolated from cells using the QuickEasy^TM^ Cell Total RNA Isolation Kit (foregene, China) according to the manufacturer's instructions. The extracted RNA was reverse transcribed into cDNA using the One Step SYBR PrimeScript RT-PCR Kit II (Takara, Japan). RT-qPCR was performed on the StepOne^TM^ and StepOnePlus^TM^ Real-Time PCR system under manufacturer-recommended conditions. The β-actin served as the endogenous control, and relative quantification was performed using the 2^-ΔΔCt method. RT-qPCR primers used were detailed in Table S2 of the supplementary file.

### Wound healing assay

B16 cells in the logarithmic growth phase were adjusted to a concentration of 5×10^5^ cells/mL in the shNC and shKRT17 groups. 2 mL of cells were seeded into each well of a 6-well plate, with three replicate wells per group, and cultured until 100% confluency was reached. Create a wound by manually scraping the cell monolayer perpendicular to the bottom of the well with a sterile 200 μL pipette tip. Wash three times with PBS (Wuhan Pricella Biotechnology Co., Ltd, China) to remove detached cells, then add media. Photograph the same location under an inverted microscope at 0 h, 24 h, and 48h after creating the wound. Measure wound width and calculate migration rate using ImageJ software. The experiment was independently repeated three times.

### Cell proliferation assay

Adjust the concentration of cells in both groups to 1×10^4^ cells/mL during the logarithmic growth phase. Seed 100 μL per well into a 96-well plate with 6 replicates per group. Set up a blank control group and moisturize the marginal wells with PBS. Incubate at 37℃ with 5% CO_2_ for 0, 24, 48, 72, and 96 hours. At each time point, add 10 μL of Cell Counting Kit-8 (MedChemExpress, China) to each well. After incubation for 2 hours, measure the absorbance (OD value) at 450 nm wavelength using a microplate reader. Each group was independently repeated three times. Calculate the mean value, subtract the OD value of the blank control, and then plot the proliferation curve with differential analysis.

### Statistical analysis

The R software (version 4.5.1) and GraphPad Prism software were used to analyze all statistics. The *p*-value < 0.05 was considered statistically significant.

## Results

### Senescent status in SKCM Patients

To explore senescent status in SKCM, we performed t-SNE analysis on the TCGA-SKCM cohort using a senescence signature gene set comprising 124 genes. This analysis ultimately divided patients into two distinct clusters (Figure 1B). The two distinct clusters (cluster 1 and cluster 2) comprised 230 and 239 patients, respectively. Survival analysis comparisons revealed statistically significant differences between the two clusters (*p*-value < 0.001). Patients in cluster 2 exhibited significantly better OS than those in cluster 1 (Figure 1C). Furthermore, it is known that as senescence progresses, SASP promotes SKCM invasion and metastasis, leading to significantly reduced survival in elderly SKCM patients. Thus, tumor lesions in patients from cluster 1 and cluster 2 may reside in severe senescent and mildly senescent microenvironment, respectively. To validate this hypothesis, we performed differential expression analysis between the two clusters. We then used three gene sets to assess the distribution of DEGs between the two clusters across these three gene sets. Among the 12 genes down-regulated in senescent cells, 8 genes (66.7%) were highly expressed in cluster 2 but down-regulated in cluster 1 (Figure 1D). Of the 18 genes down-regulated in primary fibroblast cultures from normal old donors, 13 (72.2%) were highly expressed in cluster 2 (Figure 1E). Furthermore, although 2 of the 4 genes that trigger senescence in vitro and in vivo were upregulated in cluster2, this showed no statistical significance (*p*-value > 0.05). The remaining 2 senescence driver genes were highly expressed in cluster1, both exhibiting significant statistical differences (*p*-value < 0.01) (Figure 1F). These results indicate that the identified clusters are significantly correlated with senescent status. Therefore, we defined patients in cluster1 and cluster 2 as the high-senescence group and low-senescence group, respectively.

### Prognostic value of TIME status in SKCM patients

**Figure 2:**
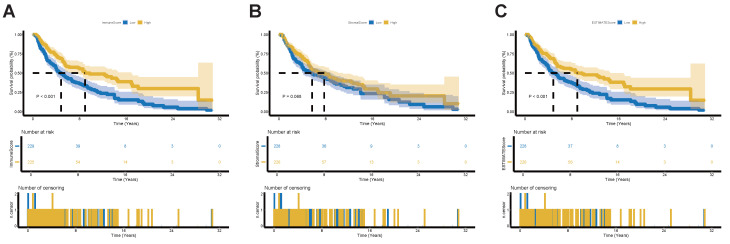
** Validation of the prognostic role of TIME status in SKCM Patients.** (A) Kaplan-Meier survival analysis for SKCM patients divided into high-score or low-score groups determined by the comparison with the median of ImmuneScore. *p*-value < 0.001 by the log-rank test. (B) Kaplan-Meier survival analysis for SKCM patients divided into high-score or low-score groups determined by the comparison with the median of StromalScore. *p*-value = 0.068 by the log-rank test. (C) Kaplan-Meier survival analysis for SKCM patients divided into high-score or low-score groups determined by the comparison with the median of ESTIMATEScore. *p*-value < 0.001 by the log-rank test. **Abbreviations**: TIME, tumor immune microenvironment.

To assess the TIME status of SKCM patients, we employed the ESTIMATE algorithm to calculate the ImmuneScore, StromalScore and the combined ESTIMATEScore, which reflects the overall proportion of immune and stromal components within TIME. Patients were then divided into high-score and low-score groups based on the median score, with higher scores indicating greater immune and stromal infiltration and lower tumor purity within TIME. Our results demonstrate that the proportion of immune components is positively correlated with OS (Figure 2A). Although StromalScore showed no significant correlation with OS, a trend toward improved OS was observed in the high-StromalScore group compared to the low-StromalScore group (Figure 2B). The ESTIMATEScore also positively correlated with OS (Figure 2C). These findings indicate that integrating immune and stromal components within TIME can serve as a prognostic indicator in SKCM patients. Based on TIME status, the high- and low-ESTIMATEScore groups reflect varying levels of immune and stromal components beyond tumor purity. For simplicity, we designate the high-ESTIMATEScore group—patients exhibiting a high TIME profile less favorable for tumor growth—as the high-immunity group.

To determine the relationship between TIME status and clinical-pathological characteristics, we analyzed corresponding clinical information for SKCM cases in the TCGA database. We found that ImmuneScore, StromalScore, and ESTIMATEScore were all negatively correlated with T classification of TNM stage (Figure S1). This suggests that the proportions of immune and stromal components are linked with SKCM progression, such as tumor infiltration and invasion. In summary, the ESTIMATE algorithm can effectively assess prognosis in SKCM patients and also shows notable correlation with clinical characteristics.

### Functional enrichment analysis of senescence-TIME-related DEGs in SKCM patients

**Figure 3:**
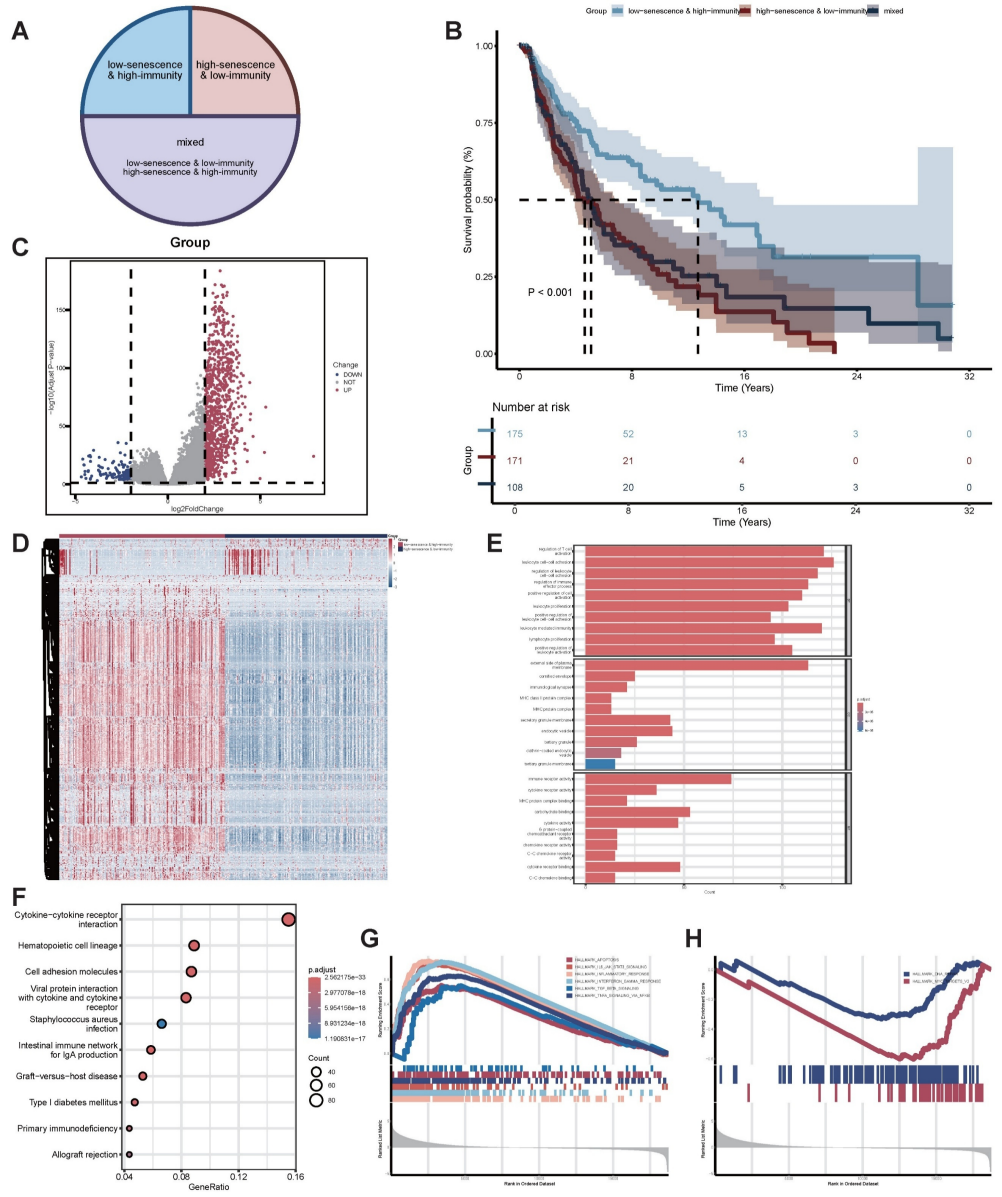
** Identification of functional enrichment analysis of senescence-TIME-related DEGs.** (A) Pie Chart of patient grouping. (B) Kaplan-Meier survival analysis for SKCM patients divided into three groups. *p*-value < 0.001 by the log-rank test. (C) Volcano plot of 994 senescence-TIME-related DEGs. (D) Heat map of 994 senescence-TIME-related DEGs. (E) Category display bar chart of GO enrichment analysis, showing the most enriched biological processes, cellular components, and molecular functions. (F) Bubble chart of KEGG enrichment analysis, showing the most enriched pathways. (G) GSEA of immunity-related pathways in the low-senescence & high-immunity group, showing significantly enriched pathways. (H) GSEA of senescence-related pathways in the high-senescence & low-immunity group, showing significantly enriched pathways. **Abbreviations**: DEGs, differentially expressed genes; GO, gene ontology; KEGG, kyoto encyclopedia of genes and genomes; GSEA, gene set enrichment analysis.

Based on the aforementioned senescent and TIME status, we further combined them into a two-dimensional metric, categorizing patients into three groups: low-senescence & high-immunity, high-senescence & low-immunity, and mixed (Figure 3A). Survival analysis revealed statistically significant differences in OS among the three groups (*p*-value < 0.001). Patients in the low-senescence & high-immunity group demonstrated the best OS, while those in the high-senescence & low-immunity group had the poorest prognosis (Figure 3B).

To identify senescence-TIME-related DEGs, we further performed comparisons of gene expression profiles between the low-senescence & high-immunity group and the high-senescence & low-immunity group. A total of 994 senescence-TIME-related DEGs were identified (Figure 3C-D). Among these senescence-TIME-related DEGs, 879 were highly expressed in the low-senescence & high-immunity group, while 115 genes were highly expressed in the high-senescence & low-immunity group. These genes were defined as senescence-TIME-related protective DEGs and senescence-TIME-related risk DEGs, respectively. Next, we performed GO, KEGG pathway analysis, and GSEA on these 994 DEGs to predict their potential functions. As expected, DEGs were predominantly enriched in immune and senescent pathways, such as leukocyte cell-cell adhesion, regulation of T cell activation, immune receptor activity, cytokine-cytokine receptor interaction, IL6-JAK-STAT3 signaling, Interferon-γ response, apoptosis, DNA repair, and so on (Figure 3E-H). Thereby, the general roles of DEGs mapped to activities relevant to senescence and immunity, suggesting these DEGs may be determinants of the senescent and TIME status. To further explore connections among these DEGs, we constructed a PPI network using Cytoscape software based on the STRING database (Figure S2A). The bar chart displays the top 80 genes ranked by node number (Figure S2B).

In summary, by integrating senescent and TIME status, we revealed their crucial roles in SKCM patient prognosis and identified a set of DEGs. These DEGs were significantly enriched in immunity- and senescence-related pathways, confirming the reliability and accuracy of the grouping. The DEGs also exhibited complex interactions through the PPI network, further supporting their potential functional roles in regulating the senescent and TIME status of SKCM.

### Construction of STIRS to predict SKCM patient prognosis using LASSO regression and RF algorithm

**Figure 4:**
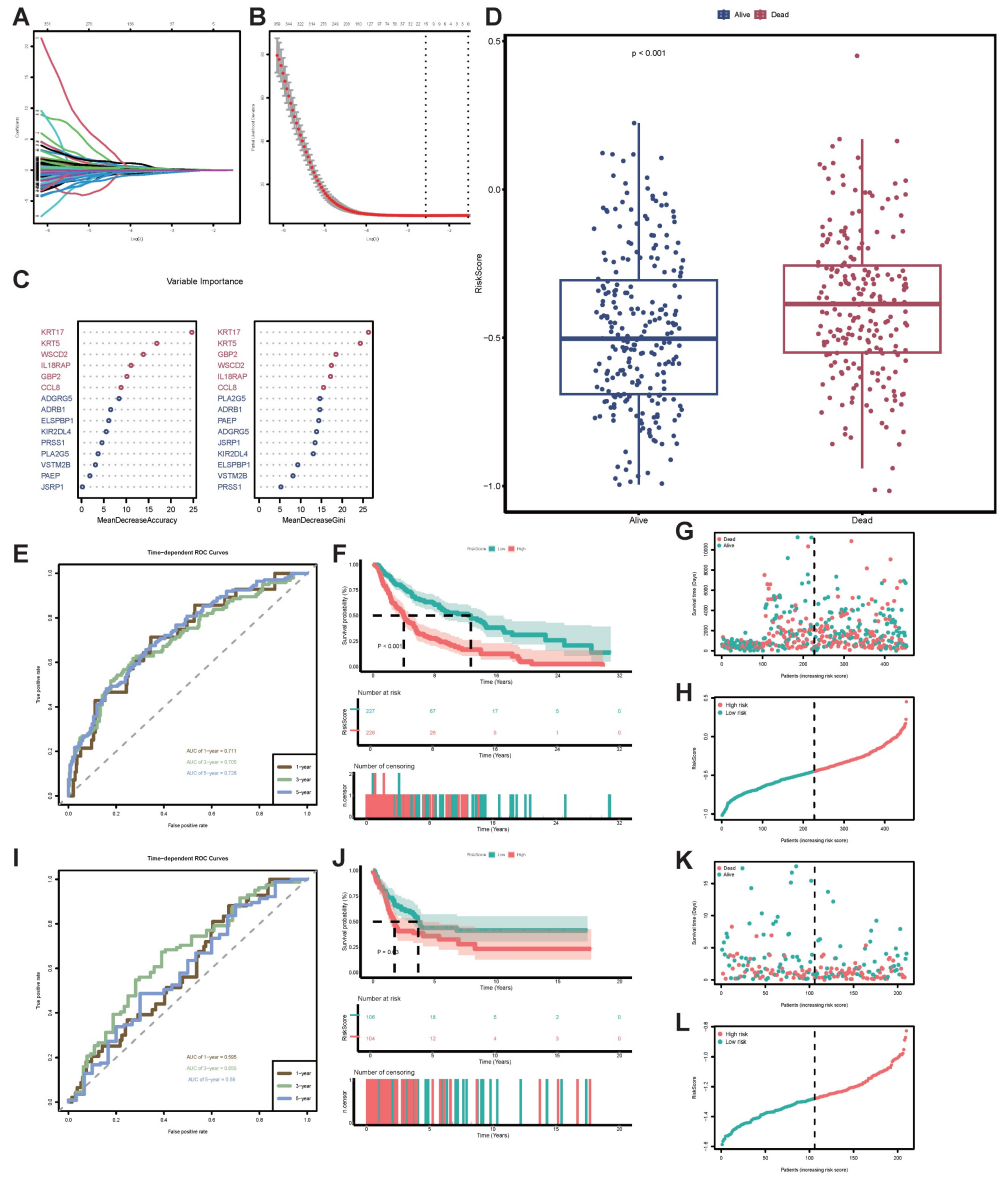
** Construction of STIRS to predict prognosis of SKCM patients.** (A) LASSO coefficient profiles for senescence-TIME-related prognostic DEGs, with coefficient values for the selected genes plotted against the penalty parameter (lambda). (B) Cross-validation curve for the LASSO regression model, used to determine the optimal penalty parameter (lambda) for the selection of prognostic genes. (C) Variable importance of screened DEGs based on mean decrease accuracy and mean decrease gini from RF algorithm. (D) Grouped bar chart of STIRS distribution in alive and dead TCGA-SKCM cases. *p*-value < 0.001 by the wilcoxon rank-sum test. (E) Time-dependent ROC curves for the STIRS at 1-, 3-, and 5-year in TCGA-SKCM dataset, with AUC values indicating the prognostic performance of the STIRS. (F) Kaplan-Meier survival analysis for TCGA-SKCM dataset patients divided into high-score or low-score groups determined by the comparison with the median of STIRS. *p*-value < 0.001 by the log-rank test. (G-H) Risk factor diagrams of high-STIRS and low-STIRS groups from TCGA-SKCM dataset. (I) Time-dependent ROC curves for the STIRS at 1-, 3-, and 5-year in GSE65904 dataset, with AUC values indicating the prognostic performance of the STIRS. (J) Kaplan-Meier survival analysis for GSE65904 dataset patients divided into high-score or low-score groups determined by the comparison with the median of STIRS. *p*-value < 0.001 by the log-rank test. (K-L) Risk factor diagrams of high-STIRS and low-STIRS groups from GSE65904 dataset. **Abbreviations**: STIRS, senescence-TIME risk score; LASSO, least absolute shrinkage and selection operation; RF, random forest; TCGA, the cancer genome atlas program; ROC, receiver operator characteristic.

To obtain key genes, we first input 994 DEGs into LASSO regression, based on lambda at minimum binomial deviation as the criterion (Figure 4A-B), thus identifying 15 candidate genes. Subsequently, we incorporated these 15 candidate genes into the RF classifier for importance ranking, ultimately selecting the top 6 genes with the highest mean decrease accuracy and mean decrease gini as the final key genes: KRT17, keratin 5 (KRT5), guanylate binding protein 2 (GBP2), WSC domain containing 2 (WSCD2), interleukin 18 receptor accessory protein (IL18RAP), C-C motif chemokine ligand 8 (CCL8) (Figure 4C). We created a unique STIRS based on the expression levels of these genes. Patients were grouped according to the median STIRS score, revealing significantly better survival outcomes in the low-STIRS group compared to the high-STIRS group (Figure 4D). This demonstrated that our risk score can predict prognosis in SKCM patients. Furthermore, the AUC for 1-, 3-, and 5-year prognosis prediction based on STIRS reached 0.711, 0.705, and 0.726, respectively, showing robust predictive value of STIRS (Figure 4E). When comparing high-risk and low-risk cohorts using Kaplan-Meier survival analysis, the high-risk cohort's OS was noticeably worse (*p*-value < 0.001) (Figure 4F). Risk factor diagrams also revealed a higher proportion of fatal cases in the high-STIRS group (Figures 4G-H).

To validate the predictive capability of this risk score, we applied STIRS to predict prognosis in SKCM patients within the validation set GSE65904. The distribution of STIRS differed between patients with varying prognoses, and the prognostic difference between high-risk and low-risk groups is statistically significant (*p*-value < 0.05) (Figure 4J-L). In the validation cohort, the AUC achieved 0.595, 0.655, and 0.59 for 1-, 3-, and 5-year OS based on STIRS, respectively, confirming its predictive value (Figure 4I). In conclusion, we successfully constructed and validated a prognostic prediction score integrating senescent and TIME characteristics. STIRS, derived from RNA-seq data analysis and machine learning, effectively predicts the clinical prognosis of SKCM patients.

### Nomogram for predicting the prognosis of SKCM patients based on STIRS and clinical indicators

**Figure 5:**
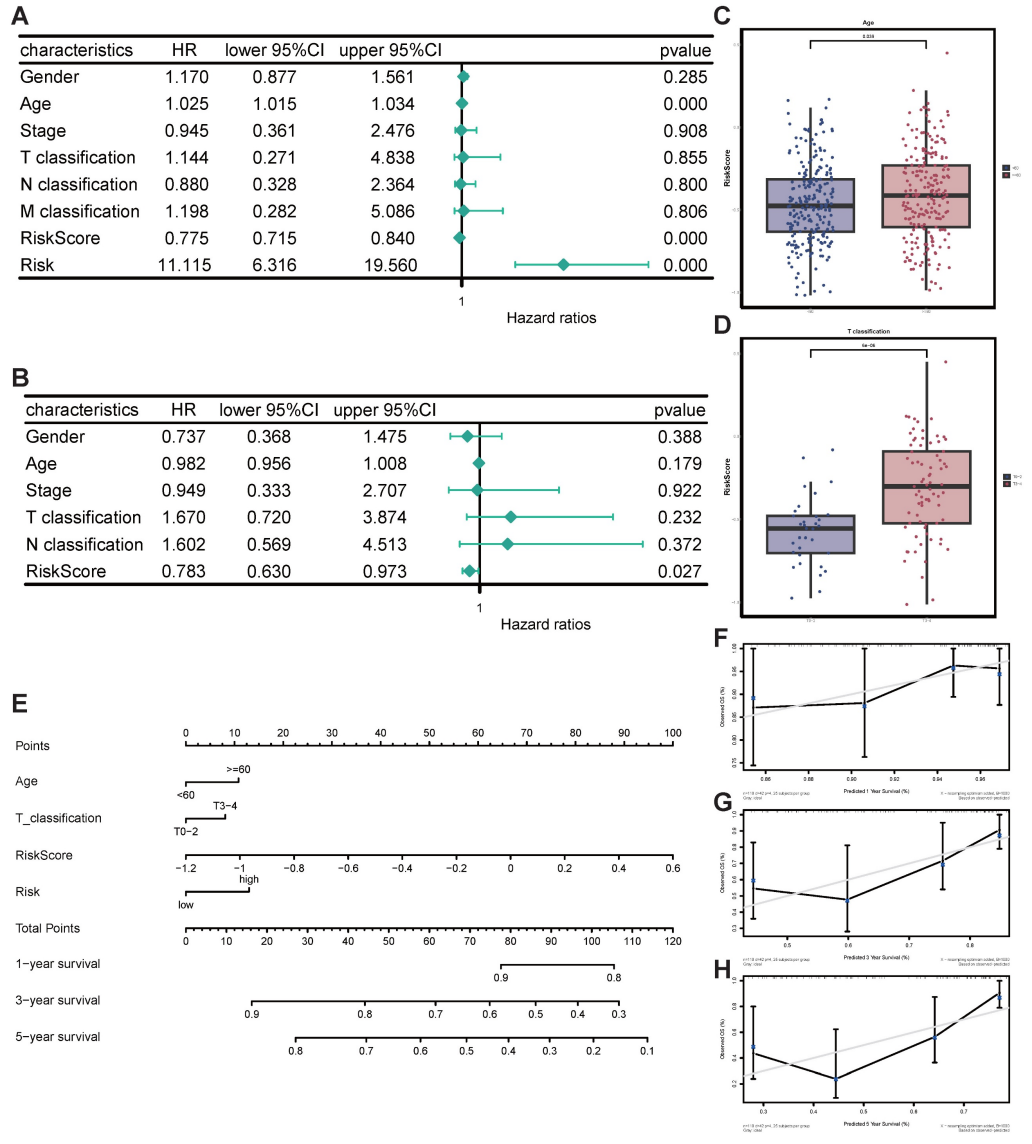
** Construction and validation of nomogram for predicting prognosis of SKCM patients.** (A) Forest plot of univariate Cox analysis. (B) Forest plot of multivariate Cox analysis. (C) Grouped bar chart of STIRS distribution in young and old patients. *p*-value = 0.039 by the wilcoxon rank-sum test. (D) Grouped bar chart of STIRS distribution in T0-2 and T3-4 patients. *p*-value < 0.001 by the wilcoxon rank-sum test. (E) Nomogram integrating STIRS and clinical pathological parameters for predicting 1-, 3-, and 5-year OS in SKCM patients. (F-H) The calibration curve for the predicted and observed 1-, 3-, 5-year OS.

First, univariate and multivariate Cox regression analyses proved the independent prognostic significance of STIRS alongside other clinical pathological parameters. Notably, univariate Cox analysis revealed that risk score (HR = 0.775, *p*-value < 0.001), risk (HR = 11.115, *p*-value < 0.001), and age (HR = 1.025, *p*-value < 0.001) were significantly associated with SKCM prognosis (Figure 5A). Additionally, multivariate Cox analysis revealed that risk score (HR = 0.783, *p*-value < 0.05) was also significantly correlated with SKCM prognosis (Figure 5B). Collectively, Cox regression analysis results indicate that STIRS represents an independent prognostic risk factor for SKCM patients. The distribution of STIRS in SKCM patients closely correlates with prognostic factors such as tumor node metastasis classification, stage, gender, and age. Patients with advanced cancer (higher T classification) and older age exhibit significantly elevated STIRS (Figure 5C-D, Figure S2C-F). Subsequently, to enhance clinical practice, we integrated STIRS with clinical pathological parameters to construct a nomogram predicting 1-, 3-, and 5-year OS of SKCM patients (Figure 5E). The accuracy and reliability of the nomogram were verified via calibration curve analysis. Results showed that the predicted curves for 1-, 3-, and 5-year OS closely aligned with observed outcomes (Figure 5F-H). Therefore, this nomogram can be applied in clinical practice to forecast survival in SKCM patients.

### The negative correlation of STIRS with immunotherapy responsiveness in SKCM patients

**Figure 6:**
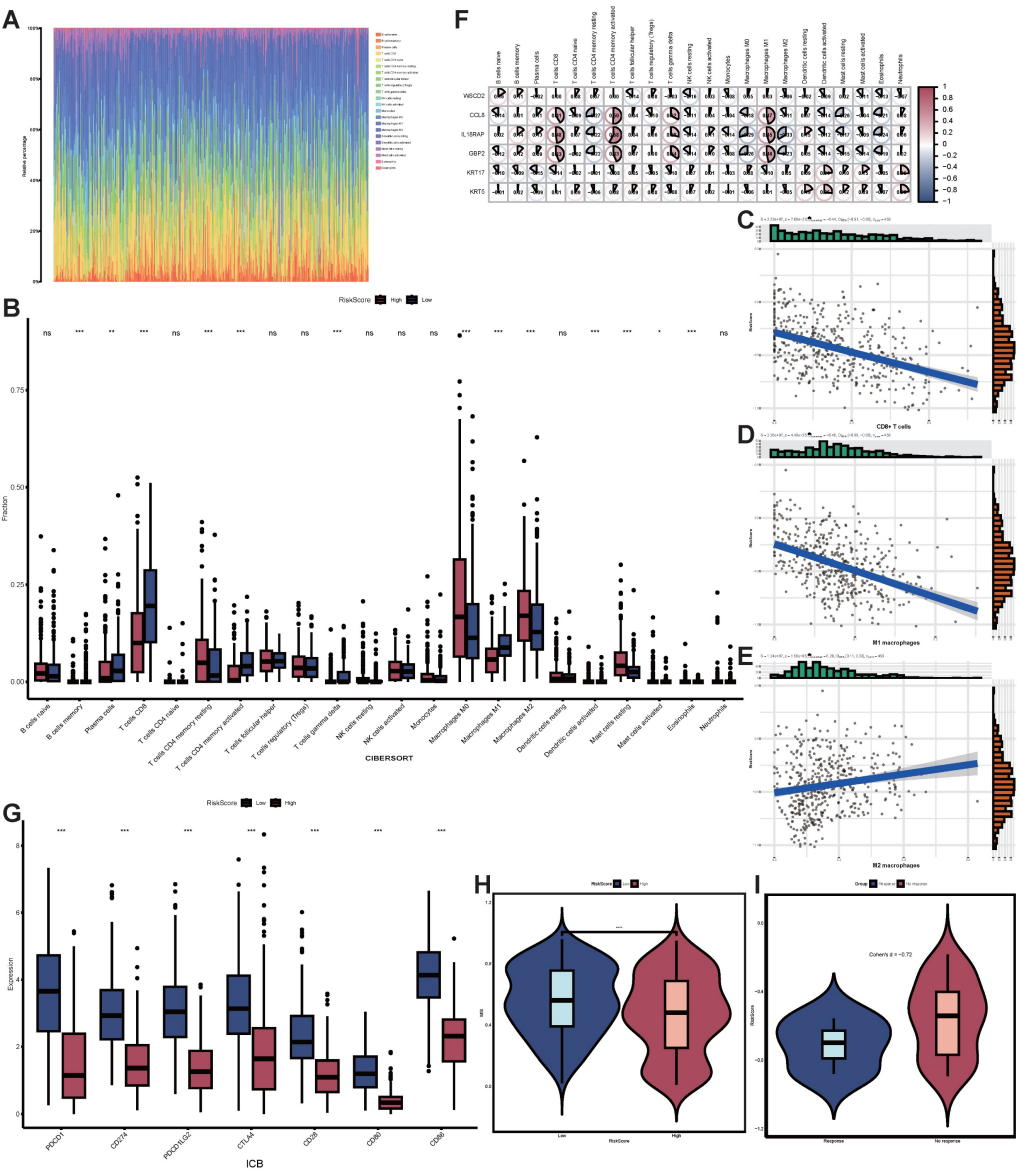
** Correlation of STIRS with immune responses and immunotherapy responsiveness.** (A) Rainbow chart of 22 immune cell types using the CIBERSORT algorithm. (B) Grouped bar chart of the proportion of 22 immune cell types in low-STIRS group and high-STIRS group. ns, *p*-value > 0.05; *, *p*-value < 0.05; **, *p*-value < 0.01; ***, *p*-value < 0.001 by the wilcoxon rank-sum test. (C-E) Correlation scatter plot of STIRS and CD8^+^ T cells, M1 macrophages, and M2 macrophages. (F) Correlation heat map of the 6 genes constituting STIRS and 22 immune cell types. (G) Grouped bar chart of primary immune checkpoints in low-STIRS group and high-STIRS group. ns, *p*-value > 0.05; *, *p*-value < 0.05; **, *p*-value < 0.01; ***, *p*-value < 0.001 by the wilcoxon rank-sum test. (H) The distribution MSI scores in low-STIRS group and high-STIRS group. ****, *p*-value < 0.0001 by the t test. (I) The effect size of STIRS differences between responders and non-responders received ICIs treatment from GSE115821 dataset. **Abbreviations**: CIBERSORT, cell-type identification by estimating relative subsets of RNA transcripts; MSI, microsatellite instability; ICIs, immune checkpoint inhibitors.

To better comprehend the correlation between STIRS and tumor immunity, we investigated the infiltration of 22 immune cell types using the CIBERSORT algorithm (Figure 6A, Figure S3A). The grouped bar chart revealed that the low-STIRS group exhibited significantly higher proportions of CD8^+^ T cells and M1 macrophages compared to the high-STIRS group, while harboring significantly fewer M2 macrophages (Figure 6B). ssGSEA analysis corroborated the CIBERSORT findings (Figure S3B). Further analysis revealed that STIRS negatively correlated with the proportions of CD8^+^ T cells and M1 macrophages, while positively correlating with the proportion of M2 macrophages (Figures 6C-E, Figure S3C-K). It is well known that high expression of CD8^+^ T cells and M1 macrophages suppresses tumor progression, whereas M2 macrophages suppress T cell-mediated antitumor immune responses to create a permissive environment for tumor growth and metastasis. Therefore, we conclude that STIRS negatively correlates with immune responses. Furthermore, we performed correlation analyses between the 6 genes constituting STIRS and 22 immune cell types, revealing that CCL8, IL18RAP, and GBP2 showed significantly stronger immunological correlations (Figure 6F). This suggested their critical roles in tumor immunity.

Based on these findings, we further explored the clinical utility of STIRS in predicting immunotherapy responsiveness in SKCM patients. Results showed that key immune checkpoints such as PD-1 and PD-L1 exhibited significantly higher expression levels in the low-risk group compared to the high-risk group (*p*-value < 0.001), suggesting that SKCM patients with low STIRS may respond better to ICIs treatment (Figure 6G). To validate this hypothesis, we employed the TIDE algorithm to assess the potential therapeutic efficacy of immunotherapy in different groups. In the TCGA-SKCM dataset, the high-risk group exhibited significantly lower MSI scores (p < 0.0001), suggesting increased immune escape potential in high-STIRS patients, which may reduce their sensitivity to ICIs (Figure 6H). Furthermore, in the GSE115821 dataset containing immunotherapy responsiveness data, the effect size of STIRS differences between responders and non-responders was substantial (Cohen's d = -0.72), suggesting STIRS could serve as an indicator for assessing immunotherapy efficacy (Figure 6I). Collectively, these findings indicate the potential clinical utility of STIRS in predicting immunotherapy benefit among SKCM patients, providing important theoretical support for developing personalized immunotherapy strategies.

### The role of KRT17 in promoting SKCM progression

**Figure 7:**
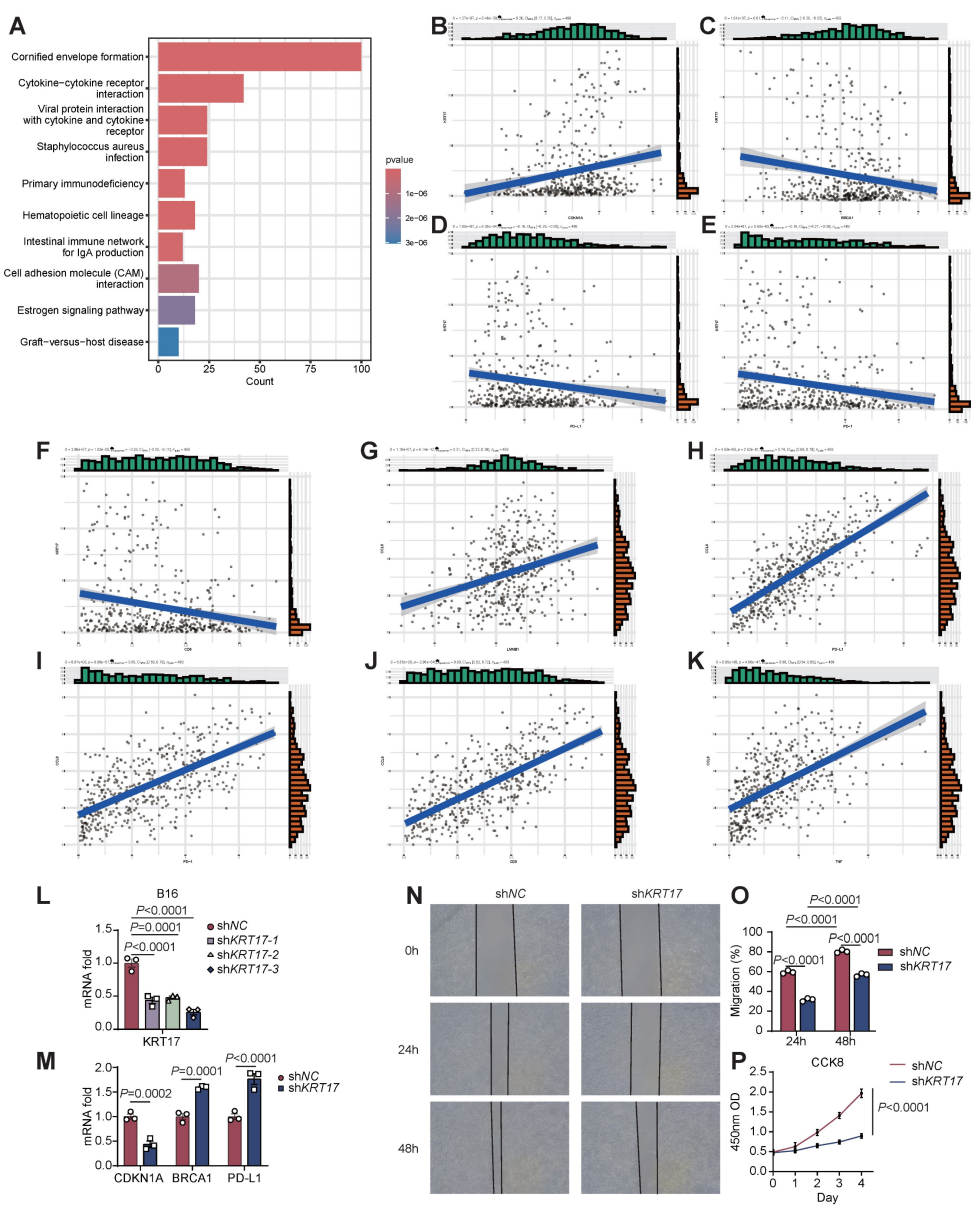
** Validation of KRT17-mediated SKCM progression.** (A) Bar chart of KEGG enrichment analysis, showing the most enriched pathways. (B-F) Correlation scatter plot of KRT17 and CDKN1A, BRCA1, PD-L1, PD-1, and CD8. (G-K) Correlation scatter plot of CCL8 and LMNB1, PD-L1, PD-1, CD8 and TNF. (L) The mRNA expression level of KRT17 in B16 cells transfected with shNC and three shKRT17 sequences, detected by RT-qPCR (n=3), with statistical significance assessed using ANOVA. (M) The mRNA expression levels of CDKN1A, BRCA1, and PD-L1 in shNC and shKRT17 groups, detected by RT-qPCR (n=3), with statistical significance assessed using ANOVA. (N-O) Wound healing of B16 cells in shNC and shKRT17 groups at 0h, 24h, and 48h, and quantification of cell migration rates at 24h and 48h (n=3), statistical significance assessed using ANOVA. (P) Proliferation capacity of B16 cells in shNC and shKRT17 groups from day 0 to 4, detected by CCK8 (n=3), statistical significance assessed using ANOVA. **Abbreviations**: KRT17, keratin 17; CDKN1A, cyclin-dependent kinase inhibitor 1A; BRCA1, breast cancer susceptibility gene 1; PD-L1, programmed cell death ligand 1; PD-1, programmed cell death protein 1; CCL8, C-C motif chemokine ligand 8; LMNB1, lamin B1; TNF, tumor necrosis factor; RT-qPCR, quantitative reverse transcription polymerase chain reaction; ANOVA, analysis of variance; CCK8, Cell Counting Kit-8.

Among the six signature genes constituting STIRS, KRT17 and KRT5 are risk genes, exhibiting elevated expression in patients with poorer prognosis, while the remaining four genes are protective genes. To further investigate the specific roles of these six signature genes in promoting SKCM progression, patients were stratified into high-STIRS and low-STIRS groups based on the median STIRS score. KEGG enrichment analysis of DEGs between groups revealed that the cornified envelope formation pathway included KRT5 and KRT17 genes, while the cytokine-cytokine receptor interaction pathway and viral protein interaction with cytokine and cytokine receptor pathway included CCL8 and IL18RAP genes (Figure 7A). Additionally, GSEA revealed that genes highly expressed in the low-STIRS group were predominantly enriched in immunity-related pathways, with the interferon-α (IFN-α) pathway containing the GBP2 gene (Figure S4A). These results indicated that these signature genes are associated with immunity and SKCM development.

Further correlation analyses of these signature genes with senescence- and immunity-related genes showed that KRT17 positively correlated with the pro-senescence gene cyclin-dependent kinase inhibitor 1A (CDKN1A), negatively correlated with the anti-senescence gene breast cancer susceptibility gene 1 (BRCA1), and inversely correlated with PD-L1, PD-1, and CD8 expression (Figure 7B-F). CCL8 exhibited the highest coefficient among the 6 genes constituting STIRS, positioning it as the most central protective gene. CCL8 showed positive correlations with the anti-senescence gene LMNB1 and with PD-L1, PD-1, tumor necrosis factor (TNF), and CD8 (Figure 7G-K). Similarly, protective genes IL18RAP and GBP2 showed correlations with senescence- and immunity-related genes consistent with the results of CCL8 (Figure S4B-K). These correlation analyses revealed that the risk gene KRT17 may promote SKCM progression by suppressing CD8^+^ T cell infiltration and inducing a senescent microenvironment, while low PD-1 and PD-L1 expression leads to poor response to anti-PD-1/PD-L1 therapy and worse prognosis. Protective genes, conversely, activate tumor immunity and may enhance anti-PD-1/PD-L1 treatment efficacy.

KRT17 is recognized as a prognostic marker in multiple tumors, but its role in SKCM progression remains mechanistically unexplored. Our analysis revealed that KRT17 may be associated with senescence and immunity. Consequently, we knocked down KRT17 in the B16 cell line, validated the knockdown efficiency, and selected the most efficiently downregulated cell line for subsequent experiments (Figure 7L). We validated the association between KRT17 and senescence- and immunity-related genes via RT-qPCR, with results consistent with bioinformatics analysis (Figure 7M). Subsequently, we performed wound healing assays and cell proliferation assays on both wild-type and KRT17-knockdown B16 cells. We observed reduced migration rates and slowed proliferation in KRT17-knockdown B16 cells, indicating that KRT17 may promote SKCM progression also by enhancing tumor cell invasion and proliferation, thereby contributing to poorer patient prognosis (Figure 7N-P). These findings suggested that KRT17 holds promise as a potential clinical target for SKCM intervention.

## Discussion

Given the substantial variability in SKCM prognosis, establishing a reliable and stable scoring system for patient stratification is crucial for maximizing the benefits of personalized treatment and timely follow-up. Consequently, considerable efforts have been devoted to exploring the complex mechanisms underlying SKCM. However, current understanding of prognostic factors and immunotherapy remains far from satisfactory. This study aims to comprehensively explore senescent and TIME characteristics of RNA-seq to develop a tool that addresses this critical clinical issue.

The tumor cellular senescence and immune microenvironment play crucial roles in SKCM tumorigenesis and progression. On one hand, profound immune suppression within TIME constitutes a key mechanism for tumor escape from host immunity and a primary reason for the limited clinical efficacy of current immunotherapies 25. On the other hand, tumor cellular senescent factors such as components of SASP within the microenvironment contribute to establishing a pro-inflammatory and immunosuppressive environment that promotes tumor cell growth, driving tumor invasion and metastasis 26. More importantly, it has been reported that the efficacy of tumor immunotherapy is compromised by the accumulation of senescent cells 27. These findings align with our research outcomes. We employed the nonlinear clustering method t-SNE to identify distinct senescent microenvironment patterns based on a gene set comprising 124 senescence marker genes, and utilized the ESTIMATE algorithm to predict the levels of infiltrating immune and stromal cells. Through these methods, we discovered that both senescent and TIME status correlate with survival in SKCM patients. Therefore, we grouped the senescent and TIME status within the TCGA-SKCM cohort, analyzed DEGs between groups, and constructed STIRS based on senescence markers and the ESTIMATE algorithm. STIRS demonstrated excellent performance in predicting prognosis of SKCM patients. When applied to a validation set, it also yielded favorable results. Subsequently, we combined STIRS with clinical parameters to construct a nomogram, which also demonstrated great performance in predicting SKCM patient prognosis. Surprisingly, STIRS not only excelled in prognosis prediction but also showed potential utility in predicting ICIs responsiveness, providing new theoretical support for precision prognosis and personalized therapy.

Although previous studies have combined senescence and immunity to predict SKCM prognosis, these analyses simply applied univariate Cox regression based on survival status and OS data to identify senescence- and immunity-related genes in datasets 28. Such methods fail to account for gene interactions and may overlook crucial polygenic effects 29. Moreover, these approaches ignore the impact of stromal components on immune responses. In contrast, t-SNE effectively captures nonlinear structures in high-dimensional data, revealing complex senescent patterns in SKCM 30. Furthermore, the ESTIMATE algorithm provides a comprehensive assessment of immune and stromal cell infiltration levels 19. When combined with senescent status, this method offers a more holistic view of the tumor immune microenvironment (TIME). By categorizing patients into distinct groups, genes associated with prognosis can be identified with greater precision, particularly those functioning within specific senescent and TIME status. To enhance model construction, we employed feature selection methods from two distinct algorithms—LASSO (linear) and RF (tree-based nonlinear)—to screen 6 genes from all differentially expressed genes (DEGs). This multi-pronged approach yields a more robust model.

Some of the key genes identified in this study have previously been reported to play important roles in multiple tumors. For example, KRT17, acting as a tumor promoter, regulates proliferation, migration, and invasion in pancreatic cancer through the mTOR/S6k1 pathway, thereby promoting pancreatic cancer progression 31. Furthermore, KRT17 gene silencing attenuates oral squamous cell carcinoma stemness, and KRT17 knockdown significantly inhibits tumor growth 32. Compared to normal tissues, IL18RAP expression is lower in hepatocellular carcinoma tissues and positively correlates with multiple anti-tumor immune cells 33. However, differing from the aforementioned studies, conflicting evidence regarding the role of IL18RAP in tumor regulation has also emerged. It was reported that IL18RAP is overexpressed in CAFs derived from gastric cancer (GC) tissues, promoting the pro-tumor function of CAFs in GC 34. The IL-18R1-IL-18RAP heterodimer can also trigger NF-κB-dependent gene expression, mediating immune escape to promote tumor progression 35. However, the mechanisms linking these signature genes to SKCM initiation and progression remain unreported, and they have rarely been studied in the combined context of TIME and senescence. Furthermore, while the preliminary roles of KRT5, GBP2, and CCL8 in SKCM progression have been elucidated, deeper molecular mechanisms remain to be explored 36, 37. WSCD2 has been identified as a prognostic factor in multiple tumors, but its specific function within oncogenesis has not been investigated 38-40. As a result, the signature genes determined in this study may provide promising targets for laboratory experimental design to clarify the molecular mechanisms of SKCM development.

Although our study demonstrates that STIRS can serve as an effective tool for predicting SKCM prognosis and immunotherapy responsiveness, several limitations require addressing. First of all, future research should utilize larger clinical cohorts to further validate the stability and generalizability of the STIRS model. Moreover, although we validated the function of the risk gene KRT17 and its association with senescence- and immunity-related genes, the specific molecular mechanisms require further experimental verification. Future studies should focus on elucidating the roles of the signature genes used to construct STIRS in SKCM progression and their impact on ICIs treatment, which needs additional basic experiments to validate the specific molecular mechanisms involved. Last but not least, researchers should evaluate the potential of these signature genes for clinical gene therapy combined with ICIs for SKCM, assess treatment prospects and long-term therapeutic results in animal models, and provide new clinical adjunct tactics for SKCM immunotherapy.

## Supplementary Material

Supplementary figures and tables, methods.

## Data Availability

The datasets presented in this study can be found in online repositories. The names of repositories and accession numbers can be found in the article.
