# Supplementary material for: Development and Validation of Novel Senescence-TIME Biomarkers for Predicting the Prognosis and Immunotherapy Responsiveness of SKCM Patients
Source: Int J Med Sci. 2026 Feb 26;23(4):1209–24. doi: 10.7150/ijms.127285 (PMC13048859; doi:10.7150/ijms.127285)
Supplement: Supplementary file 1 — Supplementary figures and tables, methods. [file ijmsv23p1209s1.pdf]

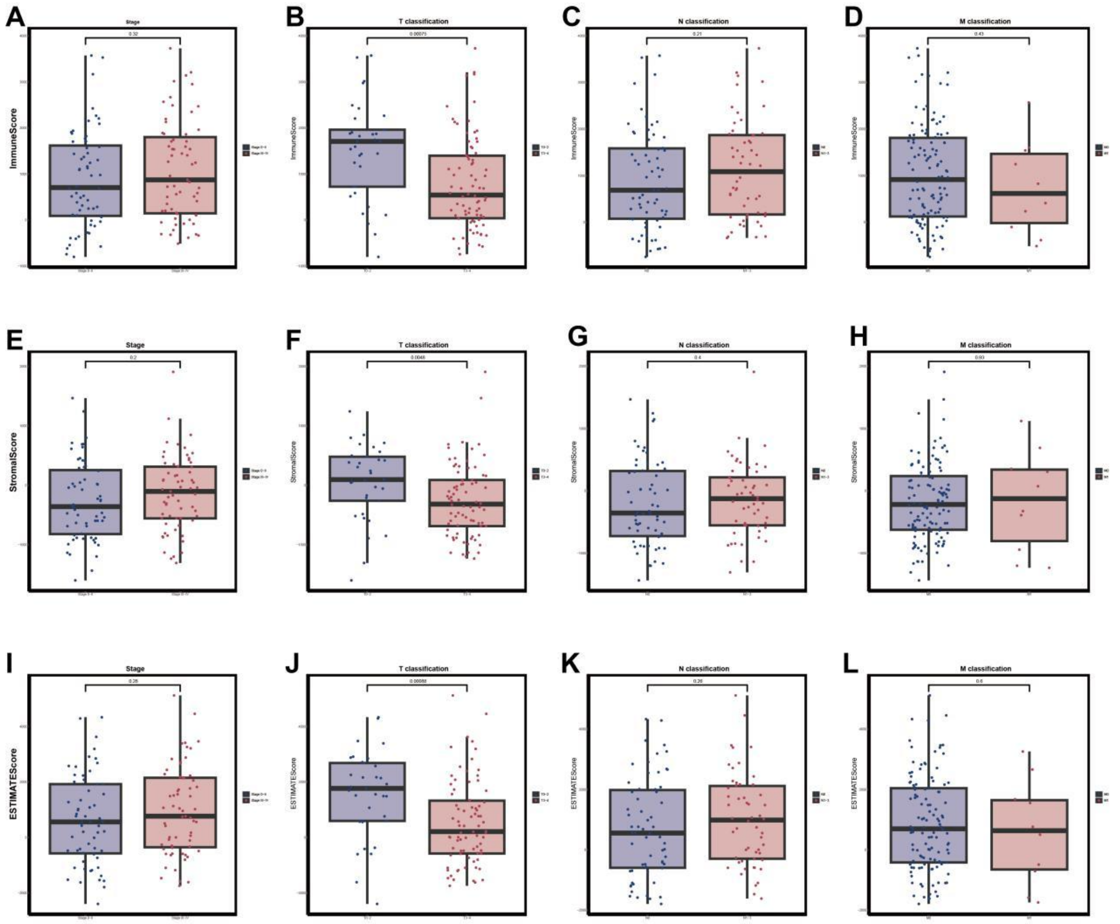

Supplementary Figure 1: Correlation of ImmuneScore, StromalScore, and ESTIMATEScore with clinical pathological parameters in SKCM patients. (A-D) Distribution of ImmuneScore in stage, T classification, N classification, and M classification. *p*-value = 0.32, 0.00075, 0.21, 0.43, respectively, by wilxcon rank-sum test. (E-H) Distribution of StromalScore in stage, T classification, N classification, and M classification. *p*-value = 0.2, 0.0048, 0.4, 0.93, respectively, by wilxcon rank-sum test. (I-L) Distribution of ESTIMATEScore in stage, T classification, N classification, and M classification. *p*-value = 0.28, 0.00088, 0.26, 0.6, respectively, by wilxcon rank-sum test.

Abbreviations: SKCM, skin cutaneous melanoma; ESTIMATE, estimation of stromal and immune cells in malignant tumor tissues using expression data.

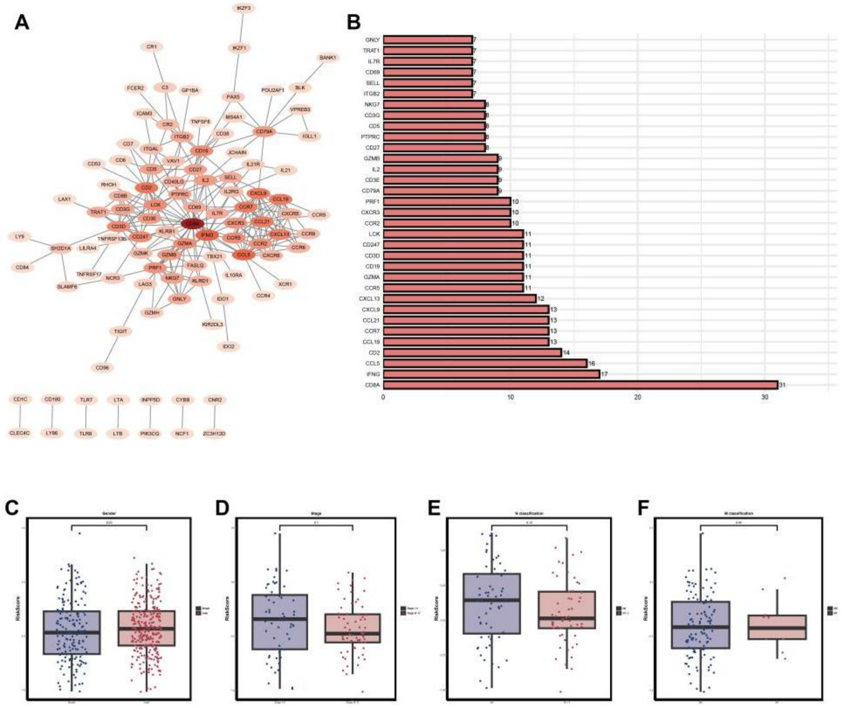

Supplementary Figure 2: Construction of PPI network and correlation of STIRS with clinical pathological parameters in SKCM patients. (A) PPI network of senescence-TIME-related DEGs. (B) Bar chart of the top 80 DEGs ranked by node number. (C) Grouped bar chart of STIRS distribution in female and male patients. *p*-value = 0.23 by the wilcoxon rank-sum test. (D) Grouped bar chart of STIRS distribution in stage I-II and stage III-IV patients. *p*-value = 0.1 by the wilcoxon rank-sum test. (E) Grouped bar chart of STIRS distribution in N0 and N1-3 patients. *p*-value = 0.12 by the wilcoxon rank-sum test. (F) Grouped bar chart of STIRS distribution in M0 and M1 patients. *p*-value = 0.95 by the wilcoxon rank-sum test.

Abbreviations: PPI, protein-protein interaction; STIRS, senescence-TIME risk score; DEGs, differentially expressed genes.

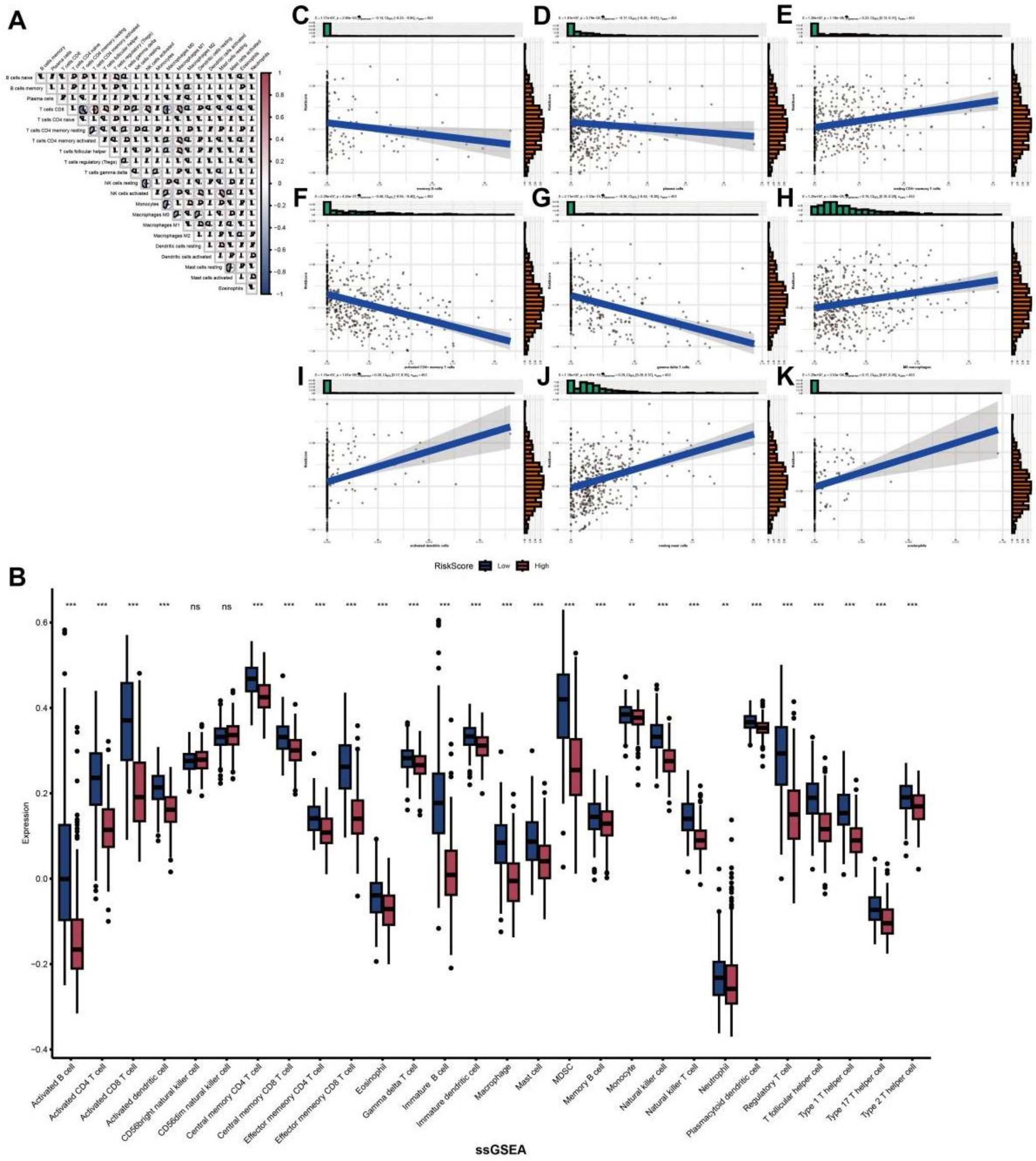

Supplementary Figure 3: Correlation of STIRS with immune infiltration in SKCM. (A) Correlation heat map of 22 immune cell types. (B) Grouped bar chart of the expression of 28 immune cell types in low-STIRS group and high-STIRS group. ns, *p*-value > 0.05; *, *p*-value < 0.05; **, *p*-value < 0.01; ***, *p*-value < 0.001 by the wilcoxon rank-sum test. (C-K) Correlation scatter plot of STIRS and memory B cells, plasma cells, resting $CD4^+$ memory T cells, activated $CD4^+$ memory T cells, γδ T cells, M0 macrophages, activated dendritic cells, resting mast cells, and eosinophils.

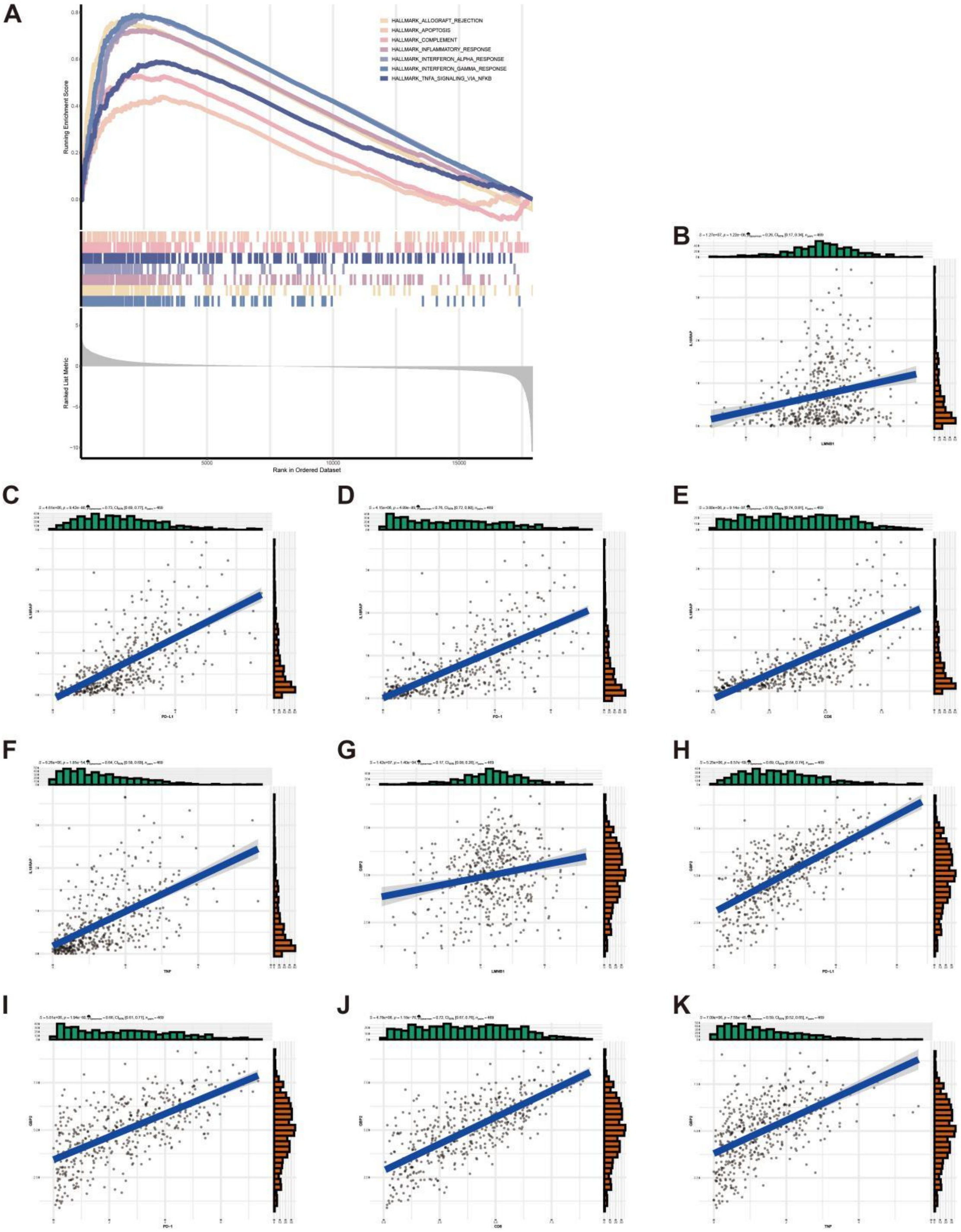

Supplementary Figure 4: Correlation of signature genes with senescence- and immunity-related pathways or genes. (A) GSEA of senescencec- and immunity-related pathways in the low-STIRS group, showing significantly enriched pathways. (B-F) Correlation scatter plot of IL18RAP and LMNB1, PD-L1, PD-1, CD8 and TNF. (G-K) Correlation scatter plot of GBP2 and LMNB1, PD-L1, PD-1, CD8 and TNF.

Abbreviations: GSEA, gene set enrichment analysis; IL18RAP, interleukin 18 receptor accessory

protein; LMNB1, lamin B1; PD-L1, programmed cell death ligand 1; PD-1, programmed cell death protein 1; TNF, tumor necrosis factor; GBP2, guanylate binding protein 2.

Table S1: The shRNA sequence targeting KRT17.

No.	Name	Oligo Sequence
1	M-Krt17-i1F	gatccGCAGTGGTTATGGAGGCAACTtcaagagAGTTGCCTCCATAACCACTGCtttttt
	M-Krt17-i1R	aattaaaaaaGCAGTGGTTATGGAGGCAACTctcttgaAGTTGCCTCCATAACCACTGCg
2	M-Krt17-i2F	gatccGGAGGTGAAGATCCGAGATTGtcaagagCAATCTCGGATCTTCACCTCCtttttt
	M-Krt17-i2R	aattaaaaaaGGAGGTGAAGATCCGAGATTGctcttgaCAATCTCGGATCTTCACCTCCg
3	M-Krt17-i3F	gatccGCGCTTATTACCATACCATTGtcaagagCAATGGTATGGTAATAAGCGCtttttt
	M-Krt17-i3R	aattaaaaaaGCGCTTATTACCATACCATTGctcttgaCAATGGTATGGTAATAAGCGCg

Table S2: RT-qPCR primers of senescence- and immunity-related genes

Primer	Sequences (5' to 3')
M-KRT17-F	CTGCTGGATGTGAAGACAAGGC
M-KRT17-R	GGTTCTTTTGGCTTGTACTGAGTC
M-CDKN1A-F	TCGCTGTCTTGCACTCTGGTGT
M-CDKN1A-R	CCAATCTGCGCTTGGAGTGATAG
M-BRCA1-F	CGAGGAAATGGCAACTTGCCTAG
M-BRCA1-R	TCACTCTGCGAGCAGTCTTCAG
M-CD274-F	TGCGGACTACAAGCGAATCACG
M-CD274-R	CTCAGCTTCTGGATAACCCTCG
M-PDCD1-F	CGGTTTCAAGGCATGGTCATTGG
M-PDCD1-R	TCAGAGTGTCGTCCTTGCTTCC
M-ACTIN-F	CATTGCTGACAGGATGCAGAAGG
M-ACTIN-R	TGCTGGAAGGTGGACAGTGAGG

Methods and materials

1. Data collection and processing

RNA-seq data and clinical information from 473 SKCM patients were downloaded from TCGA. Data from 469 cases of both primary and metastatic tumors were retained. The gene expression omnibus (GEO) database was the source of the GSE65904 and GSE115821 datasets. Transcripts per million (TPM) underwent log2 conversion.

2. Survival analysis

The R package "survminer" was utilized to do survival analysis. The Kaplan-Meier method was used to plot survival curves, and log-rank tests were used to determine statistical significance: p-value < 0.05 was deemed significant.

3. Identification of senescent status

The t-distributed stochastic neighbor embedding (t-SNE) algorithm was applied to infer the senescent status of tumor tissues in the TCGA-SKCM dataset [22]. The t-SNE is a nonparametric, unsupervised method that divides patients into distinct clusters based on given features [22]. The senescence signature gene set (genes upregulated in senescent cells: SASP), comprising 124 genes, was downloaded from MsigDB. Groups were classified as "mild senescent microenvironment" or "severe senescent microenvironment" based on survival differences between clusters. Further analysis examined expression changes in other genes involved in senescence-related regulatory processes between the two groups, including 12 genes down-regulated in senescent cells, 18 genes down-regulated in primary fibroblast cultures from normal old donors, and 4 genes that trigger senescence in vitro and in vivo, to explore the relationship between the divided groups and senescence.

4. Identification of TIME status

The R package "estimate" was used to calculate the ImmuneScore, StromalScore, and ESTIMATEScore for 469 tumor samples. Subsequently, the 453 samples with survival data were divided into high-score and low-score groups based on the median score, and survival differences between the two groups were compared.

5. Identification of senescence-TIME-related groups and differentially expressed genes (DEGs) between groups

Further combining the aforementioned senescent and TIME status into a two-dimensional metric, patients were categorized into three groups: low-senescence & high-immunity, high-senescence & low-immunity, and mixed. The gene expression profiles of the low-senescence

& high-immunity group and the high-senescence & low-immunity group were compared using the R package "DESeq2" to identify 994 senescence-TIME-related DEGs (|log2FC| > 2, FDR-adjusted p-value < 0.001).

6. Gene ontology (GO) enrichment analysis, kyoto encyclopedia of genes and genomes (KEGG) enrichment analysis, gene set enrichment analysis (GSEA), and the construction of protein-protein interaction (PPI) network

GO enrichment analysis, KEGG enrichment analysis, and GSEA were performed using the R package "clusterProfiler" to explore the functions of senescence-TIME-related genes. The PPI network was constructed using the STRING database and reconstructed using Cytoscape version 3.10.3.

7. Establishment and validation of STIRS

The least absolute shrinkage and selection operation (LASSO) regression was applied using the R package "glmnet" to screen 15 genes from 994 DEGs. To further simplify the model, random forest (RF) algorithm was conducted using the R package "randomForest". Ultimately, the top 6 genes with the highest mean decrease accuracy and mean decrease gini were selected as the final key genes for constructing the STIRS. STIRS = $\sum$(coef × mRNA expression), where coef represents the coefficients derived from LASSO. Using the R package "pROC", we calculated the area under the receiver operator characteristic (ROC) curve (AUC). STIRS was validated using the external dataset GSE65904.

8. Construction and validation of the prognostic nomogram for SKCM

Univariate and multivariate Cox regression analyses identified indicators associated with overall survival (OS). Using R package "survival" and "rms", nomogram was constructed based on STIRS and relevant clinical parameters to estimate 1-, 3-, and 5-year survival probabilities. The calibration curve was used to assess the model's performance.

9. Immune infiltration analysis

Cell-type identification by estimating relative subsets of RNA transcripts (CIBERSORT)

analysis was performed using the R package "CIBERSORT" to assess the percentage of 22 immune cell types in each SKCM patient [23]. Single sample GSEA (ssGSEA) was conducted using the R package "GSVA" to evaluate the expression of 28 immune cell types in SKCM patients.

10. Immune checkpoint expression and immunotherapy responsiveness analysis

Samples were stratified into high-STIRS and low-STIRS groups based on the median STIRS score. Expression levels of immune checkpoints, including programmed cell death protein 1 (PD-1), programmed cell death ligand 1 (PD-L1), and cytotoxic T lymphocyte-associated antigen-4 (CTLA-4), were quantified in both groups. Additionally, the microsatellite instability (MSI) score in the tumor immune dysfunction and exclusion (TIDE) system was calculated using the online platform (http://tide.dfci.harvard.edu/) to assess potential response to ICIs [24]. Differences in the response to ICIs were compared between the high-STIRS and low-STIRS groups in the GSE115821 dataset [21].

11. Correlation of signature genes with senescence- and immunity-related genes

The expression profiles of signature genes constituting STIRS, as well as senescence- and immunity-related genes in SKCM samples were extracted from the dataset. Spearman's rank correlation analysis was performed to evaluate the correlation between the expression of signature genes and the expression of senescence- and immunity-related genes.

12. Cell culture

The mouse melanoma cell line B16 and human embryonic kidney cell line 293T were obtained from Shanghai Zhong Qiao Xin Zhou Biotechnology Co., Ltd., China. Cells were cultured in DMEM (Thermo Fisher Scientific, USA) supplemented with 10% fetal bovine serum (CellMaxcell Technology, China) and maintained at 37°C in a humidified incubator with 5% CO_2.

13. shKRT17 plasmid construction and lentivirus packaging

The shKRT17 plasmid and shNC plasmid (Corues Biotechnology, China) were co-transfected with pSPAX2 and pMD2.G plasmids at a specific ratio into 293T cells using CW Plasmid

Transfection Reagent (CWBIO, China) to package lentivirus. Subsequently, B16 cells were infected with this lentiviral-mediated RNA interference system to achieve stable transfection. A 1μg/mL puromycin selection was used to screen for resistant transfected clones. Transfection efficiency was confirmed by quantitative reverse transcription polymerase chain reaction (RT-qPCR). The shRNA sequence targeting KRT17 was listed in Table S1 of the supplementary file.

14. RT-qPCR

RT-qPCR was employed to detect the expression of total RNA extracted from cultured cells. Total RNA was isolated from cells using the QuickEasy™ Cell Total RNA Isolation Kit (foregene, China) according to the manufacturer's instructions. The extracted RNA was reverse transcribed into cDNA using the One Step SYBR PrimeScript RT-PCR Kit II (Takara, Japan). RT-qPCR was performed on the StepOne™ and StepOnePlus™ Real-Time PCR system under manufacturer-recommended conditions. The β-actin served as the endogenous control, and relative quantification was performed using the 2^-ΔΔCt method. RT-qPCR primers used were detailed in Table S2 of the supplementary file.

15. Wound healing assay

B16 cells in the logarithmic growth phase were adjusted to a concentration of 5×10^5 cells/mL in the shNC and shKRT17 groups. 2 mL of cells were seeded into each well of a 6-well plate, with three replicate wells per group, and cultured until 100% confluency was reached. Create a wound by manually scraping the cell monolayer perpendicular to the bottom of the well with a sterile 200μL pipette tip. Wash three times with PBS (Wuhan Pricella Biotechnology Co., Ltd, China) to remove detached cells, then add media. Photograph the same location under an inverted microscope at 0h, 24h, and 48h after creating the wound. Measure wound width and calculate migration rate using ImageJ software. The experiment was independently repeated three times.

16. Cell proliferation assay

Adjust the concentration of cells in both groups to 1×10^4 cells/mL during the logarithmic growth phase. Seed 100 μL per well into a 96-well plate with 6 replicates per group. Set up a

blank control group and moisturize the marginal wells with PBS. Incubate at 37℃ with 5% CO_2 for 0, 24, 48, 72, and 96 hours. At each time point, add 10 μL of Cell Counting Kit-8 (MedChemExpress, China) to each well. After incubation for 2 hours, measure the absorbance (OD value) at 450 nm wavelength using a microplate reader. Each group was independently repeated three times. Calculate the mean value, subtract the OD value of the blank control, and then plot the proliferation curve with differential analysis.

17. Statistical analysis

The R software (version 4.5.1) and GraphPad Prism software were used to analyze all statistics. The *p*-value < 0.05 was considered statistically significant.
